# RETRACTED: Hsia et al. Maslinic Acid Induces Mitochondrial Apoptosis and Suppresses HIF-1α Expression in A549 Lung Cancer Cells under Normoxic and Hypoxic Conditions. *Molecules* 2014, *19*, 19892–19906

**DOI:** 10.3390/molecules30234491

**Published:** 2025-11-21

**Authors:** Te-Chun Hsia, Wen-Hu Liu, Wen-Wei Qiu, Jian Luo, Mei-Chin Yin

**Affiliations:** 1 Department of Respiratory Therapy, China Medical University, Taichung 40402, Taiwan; derrick.hsia@msa.hinet.net; 2 Department of Internal Medicine, China Medical University Hospital, Taichung 40402, Taiwan; 3 Department of Nutrition, Chung Shan Medical University, Taichung 40201, Taiwan; andy@csmu.edu.tw; 4 Institute of Medicinal Chemistry and Department of Chemistry, East China Normal University, Shanghai 200241, China; wwqiu@chem.ecnu.edu.cn; 5 Institute of Biomedical Sciences and School of Life Sciences, East China Normal University, Shanghai 200241, China; jluo@bio.ecnu.edu.cn; 6 Department of Health and Nutrition Biotechnology, Asia University, Taichung 41354, Taiwan; 7 Department of Nutrition, China Medical University, Taichung 40402, Taiwan

The Journal retracts the article “Maslinic Acid Induces Mitochondrial Apoptosis and Suppresses HIF-1α Expression in A549 Lung Cancer Cells Under Normoxic and Hypoxic Conditions” [1] cited above.

Following publication, concerns were brought to the attention of the Editorial Office regarding the integrity of Figure 2 presented in this article [1].

Adhering to our standard procedure, an investigation was conducted by the Editorial Office and Editorial Board that identified indications of inappropriate editing and partial duplications of western blots in Figure 2 presented in this article [1]. The authors were unable to satisfactorily explain the overlap of figures nor provide appropriate raw material for Editorial Board evaluation. Consequently, the Editorial Board has lost confidence in the reliability of the findings and decided to retract this publication [1], as per MDPI’s retraction policy (https://www.mdpi.com/ethics#_bookmark30).

This retraction was approved by the Editor-in-Chief of the journal *Molecules*.

The authors agreed to this retraction.
